# Modular and Substrate-Independent Grafting-To Procedure for Functional Polymer Coatings

**DOI:** 10.1021/acs.langmuir.3c00280

**Published:** 2023-05-22

**Authors:** Lucas W. Teunissen, Maarten M. J. Smulders, Han Zuilhof

**Affiliations:** † Laboratory of Organic Chemistry, Wageningen University, Stippeneng 4, Wageningen 6708 WE, The Netherlands; ‡ School of Pharmaceutical Sciences and Technology, Tianjin University, 92 Weijin Road, Tianjin 300072, People’s Republic of China

## Abstract

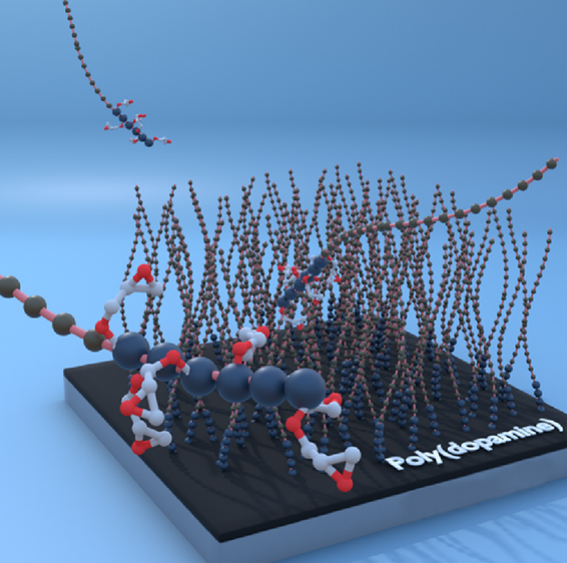

The ability to tailor polymer brush coatings to the last nanometer has arguably placed them among the most powerful surface modification techniques currently available. Generally, the synthesis procedures for polymer brushes are designed for a specific surface type and monomer functionality and cannot be easily employed otherwise. Herein, we describe a modular and straightforward two-step grafting-to approach that allows introduction of polymer brushes of a desired functionality onto a large range of chemically different substrates. To illustrate the modularity of the procedure, gold, silicon oxide (SiO_2_), and polyester-coated glass substrates were modified with five different block copolymers. In short, the substrates were first modified with a universally applicable poly(dopamine) primer layer. Subsequently, a grafting-to reaction was performed on the poly(dopamine) films using five distinct block copolymers, all of which contained a short poly(glycidyl methacrylate) segment and longer segment of varying chemical functionality. Ellipsometry, X-ray photoelectron spectroscopy, and static water contact angle measurements confirmed successful grafting of all five block copolymers to the poly(dopamine)-modified gold, SiO_2_, and polyester-coated glass substrates. In addition, our method was used to provide direct access to binary brush coatings, by simultaneous grafting of two different polymer materials. The ability to synthesize binary brush coatings further adds to the versatility of our approach and paves the way toward production of novel multifunctional and responsive polymer coatings.

## Introduction

Polymer coatings are employed ubiquitously to introduce specific surface properties required for an intended application. Control over coating properties, such as chemical functionality, polymer density, and film thickness, can be achieved especially using polymer brushes.^1−3^ This type of coating consists of end-tethered polymer chains, densely packed on a material’s surface. Generally, these polymers are either grown from surface-immobilized initiator molecules, commonly known as the grafting-from approach, or synthesized in solution and subsequently attached to the target substrate, called the grafting-to approach.^2,4^ Of the two synthesis techniques, the grafting-from procedure is most commonly employed as it allows the synthesis of thick polymer brush coatings of high grafting densities.^2,3^ The main disadvantage of this approach is related to the difficulty of characterization to determine properties such as the polymer molecular weight and polydispersity of the produced, surface-bound polymers.^5^ In contrast, the grafting-to approach allows straightforward analysis of the polymers before attachment to the surface.^4,5^ For the grafting-to approach, however, excluded-volume interactions of the polymers during the grafting step limit the number of chains that can be attached on the surface.^5^ As a result, grafting densities and layer thicknesses of polymer brushes produced via grafting-to are typically significantly lower than those produced via grafting-from.^2,5^ Yet, this limitation does also provide the ability to control grafting densities of the produced polymer brush coating by variation of the size of the grafted polymer chains, with higher grafting densities obtained for shorter polymers and *vice versa*.^5,6^

Evidently, irrespective of the employed grafting approach, the chemical and physical characteristics of the target substrate vary significantly for different materials. Due to these characteristics, the synthesis procedures for polymer brush coatings are typically substrate-dependent and cannot be applied modularly.^7,8^ For example, initiator molecules regularly employed for surface-initiated polymerizations typically contain disulfide/thiol or silane anchoring groups and are applied specifically to noble metal and hydroxyl-terminated substrates, respectively.^2,9−11^ The same consideration has to be taken into account for grafting-to procedures, in which anchoring motifs present in the presynthesized polymers are required to bind to reactive groups present on the substrate’s surface.^4^ Such complementary reactive groups are not inherently present on all surface types, and treatment with a primer layer can be necessary to introduce these.^8,12^ Naturally, to ensure a high durability of the coating, this primer layer requires a strong affinity for the substrate it is applied to.

The number of surface modification reactions that can be applied universally to any surface type is small.^7^ The procedure that has perhaps gained most traction over the past years is modification of surfaces with poly(dopamine) films.^13−16^ Inspired by mussel adhesive proteins, poly(dopamine) films have been demonstrated to successfully bind to virtually any surface type ranging from high-energy surfaces, such as noble metals and steel, to low-energy surfaces including polyethylene and polystyrene.^13^ Moreover, deposition does not require prior activation through, for example, wet etching or plasma treatment.^17−20^ The functional groups present in poly(dopamine) films, such as hydroxyl and amine groups, allow the layers to be used as primer material to perform subsequent surface modifications through formation of stable covalent bonds. In fact, the combination of broad applicability toward different surface types, procedural simplicity, and low cost of the deposition procedure has resulted in a large number of reports in which poly(dopamine) was employed to synthesize polymer brush coatings.^21−37^

Polymer brush coatings that were produced using poly(dopamine) as a primer layer were predominantly synthesized using the grafting-from approach, during which poly(dopamine) is functionalized with initiator molecules from which surface-initiated polymerization is then performed.^21,22,26,27,29−32^ In addition, there are several examples of polymers synthesized in solution and subsequently successfully grafted onto poly(dopamine) primer layers.^34−36,38^ The main advantages to this strategy are the ease of application and the possibility to fully characterize the polymers before attachment to the substrate. Moreover, grafting-to represents the most straightforward approach to produce binary and ternary polymer brush systems, i.e., polymer brushes in which several chemically different polymer chains are attached onto the surface in one step.^39−42^

Development of a standardized grafting-to procedure that can successfully bind different polymer types to poly(dopamine) primer layers would further remove a bottleneck in polymer brush synthesis methods. That procedure would allow the introduction of effectively any surface property to a substrate, irrespective of the chemical nature of the substrate or polymer. Two approaches that demonstrate modular surface modifications have been reported in recent years. The first procedure relies on one-step codeposition of dopamine with acrylate monomers that bear different functional groups.^43,44^ It was suggested that the monomers are incorporated in the poly(dopamine) structure during the polymerization of dopamine, yielding a covalent poly(dopamine)/monoacrylate network.^44^ Although this approach is appealing due to its unmatched simplicity, the presence of dopamine throughout the entire network will strongly influence the surface properties of the resulting layer. A second procedure for modular surface modification involves block copolymers carrying glycidyl methacrylate (**GMA**) units that were reacted with (3-aminopropyl)triethoxysilane (**APTES**)-functionalized substrates. As mentioned above, silane monolayers can be used to modify several types of surfaces, but cannot be employed as universally as poly(dopamine) films.^8^ The **APTES** monolayers were introduced following acid and plasma activation of the substrate and then modified with various block copolymers.^45^ Using this procedure, a broad range of surface functionalities on silicon substrates was obtained successfully and then tested extensively. Although the method worked well for silicon substrates, the modification of different substrate types revealed inconsistent surface properties, indicating that the procedure is not substrate-independent.

**Scheme 1:**
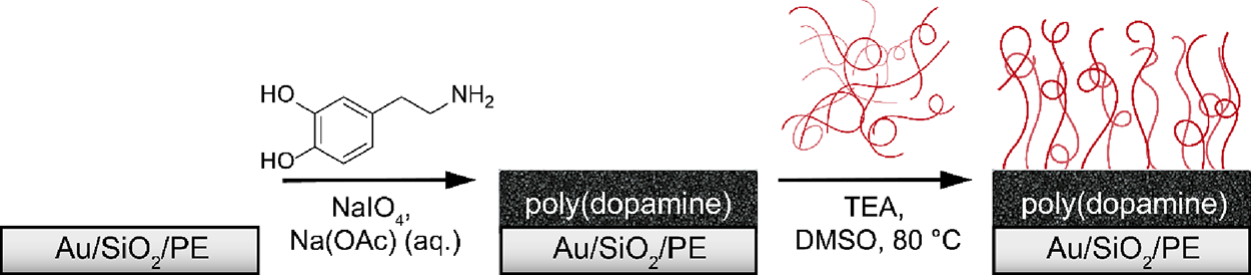
Illustration of the Grafting-To Reaction Employed in This Study A range of block copolymers (shown in red) of various compositions are grafted to poly(dopamine)-modified SiO_2_, gold, and PE substrates.

Using the insights developed in the above studies, our group developed an approach to overcome the aforementioned limitations. In previous studies, we have presented a two-step grafting-to procedure that consists of poly(dopamine) deposition on SiO_2_ followed by grafting of poly(GMA)-*b*-poly(NIPAM) block copolymers.^46,47^ In contrast to the two coating strategies described above, we demonstrated that our procedure could achieve polymer brush coatings of appreciable grafting density, and with thicknesses to 19 nm. In these studies, we demonstrated the grafting of block copolymers of varying sizes and the effect on the resulting thermoresponsive and antifouling properties of the produced poly(GMA)-*b*-poly(NIPAM) brushes.^46^ Additionally, we investigated the effect of poly(dopamine) deposition conditions on subsequent block copolymer grafting and thoroughly investigated chemical and morphological properties (including surface morphology and roughness) of the produced poly(dopamine) films and poly(GMA)-*b*-poly(NIPAM) polymer brush coatings.^47^ Yet, the procedure was exclusively performed on SiO_2_ substrates using only poly(GMA)-*b*-poly(NIPAM). In this work, we broaden the scope of that procedure and demonstrate that it can be employed modularly, irrespective of surface type or polymer functionality (Scheme 1).

To illustrate the scope of the grafting-to procedure presented in this study, three substrate types with widely varying chemical characteristics were selected as target substrates: gold (as, e.g., relevant for biomedical applications like surface plasmon resonance (SPR) sensing.^48−50^), silicon with a native silicon oxide layer (SiO_2_) (given its use in biomedical applications and photovoltaics^51,52^), and polymer-coated glass (PE), to demonstrate that the procedure can be applied to polymeric substrates, which are generally difficult to efficiently functionalize.^19^ To further illustrate the modularity of the procedure, five different block copolymers were synthesized via radical addition-fragmentation chain-transfer (RAFT) polymerization, all of which contained a short poly(glycidyl methacrylate) (poly(GMA)) segment that was incorporated to bind to the amine groups present in the poly(dopamine) film. The monomers used for the longer, second segment vary within the series of block copolymers and were selected to give rise to widely different surface properties in the final polymer coatings. The produced coatings were analyzed using spectroscopic ellipsometry, static water contact angle (SWCA) measurements, and X-ray photoelectron spectroscopy (XPS). The procedure was finally employed to simultaneously graft two different block copolymers to one substrate to achieve binary brushes, which expands the applicability of our approach for facile preparation of advanced polymer coatings even further.^39,40,53^

## Experimental Section

### Materials

All chemical reagents were used without further purification unless otherwise specified. Triethylamine (**TEA**), 2,2′-azobis(2-methylpropionitrile) (**AIBN**) (98%), poly(ethylene glycol)methyl ether methacrylate (**MeOEGMA**) (average *M*_n_ = 300, stabilized), methyl methacrylate (**MMA**) (99%, stabilized), NaOAc (>99%), and 1,4-dioxane (99.8%, anhydrous, nonstabilized) were purchased from Sigma-Aldrich. 4-Cyano-4-[(dodecylsulfanylthiocarbonyl)sulfanyl]pentanoic acid (**CDPA**) (>95.0%), dopamine.HCl (>98.0%), 2,2,2-trifluoroethyl methacrylate (**TFEMA**) (>98.0%, stabilized), and *N-*isopropylacrylamide (**NIPAM**) (>98.0%, stabilized) were purchased from TCI Chemicals Europe. Glycidyl methacrylate (**GMA**) (97%, stabilized) and methacryloyl chloride (97%, stabilized) were purchased from Thermo Fisher Scientific. NaIO_4_ (99%) was purchased from ACROS Organics. Deionized water was produced with a Milli-Q Integral 3 system Millipore, Molsheim, France (Milli-Q water). Silicon single side-polished wafers (Si(100), N-type, phosphorus-doped) were obtained from Siltronix. Silicon wafers coated with a 200 nm gold layer were acquired from Ssens. Polyester-coated glass samples were kindly provided by Tata Steel Europe, IJmuiden, Netherlands. The polyester coating was based on the monomers ethylene glycol, neopentyl glycol, isophthalic acid, phthalic anhydride, and adipic acid.

Prior to use, **GMA** was purified by washing with 0.1% m/v KOH solution and **NIPAM** was recrystallized from hexane. **MeOEGMA**, **TFEMA**, and **MMA** were passed over a basic alumina plug. **NMEP** was synthesized following a synthesis procedure described in a previous study.^40^

### RAFT Polymerization of **Poly(GMA)_20_**

The RAFT polymerization of **GMA** was performed according to a previously described procedure.^41^ The product, a fine yellow powder, was obtained through filtration and dried over air. **^1^H NMR** (400 MHz, CDCl_3_) δ 4.38–4.24 (m, 19H), 3.86–3.72 (m, 20 H), 3.26–3.19 (m, 20 H), 2.87–2.79 (m, 19H), 2.68–2.59 (m, 19 H), 2.17–1.72 (m, 34 H), 1.45–1.23 (m, 18 H), 1.11–1.07 (s, 19 H), 0.94–0.80 (s, 34 H). **IR***v* = 1740–1720 cm^–1^ (—C(=O) —O), 1470–1430 cm^–1^ (−CH_3_), 1260 cm^–1^ (C–O), 903 cm^–1^ (epoxide C–O).^59−61^

### Block Copolymerization Reactions

The polymerization reactions were carried out in an identical manner for all monomers. In short, to 100 mL Schlenk flasks were added **AIBN** (2.0 mg; 0.0023 mmol; 0.2 equiv), **poly(GMA)_20_** (32 mg; 0.012 mmol; 1 equiv), monomer (3.45 mmol; 300 equiv), and 1,4-dioxane (3 mL). The solutions were deoxygenated by three freeze-pump-thaw cycles. After the last cycle, the flask was charged with argon. The flask was then heated at 70 °C and left to stir.

The polymerization **MeOEGMA** was stopped after 4 h by removing the flask from the heat source and opening the reaction mixture to the atmosphere. To the solution was then added acetone (10 mL), after which the polymer was purified by addition to cold hexane, centrifugation and decantation of the hexane layer. A viscous, transparent, and colorless product was obtained. **^1^H NMR** (400 MHz, CDCl_3_) δ 4.08 (s, 2 H), 3.82–3.47 (m, 16 H), 3.38 (s, 3 H), 1.84 (m, 2 H), 0.95 (m, 3 H). **IR***v* = 2970–2830 cm^–1^ (C–H), 1726 cm^–1^ (—C(=O)—O), 1097 cm^–1^ (C–O–C).^62,63^

The polymerization of **NIPAM** was stopped after 4 h by removing the flask from the heat source and opening the reaction mixture to the atmosphere. To the solution was then added acetone (10 mL), after which the polymer was purified by 2-fold precipitation from cold diethyl ether and filtration. A white powder was obtained. **^1^H NMR** (400 MHz, CDCl_3_) δ 4.00 (s, 1 H), 2.58 (s, 1 H), 1.45–0.96 (m, 6 H). **IR***v* = 3297 cm^–1^ (N–H), 1640 cm^–1^ (—C(=O)—NH), 1542 cm^–1^ (N–H).^64,65^

The polymerization of **MMA** was stopped after 48 h by removing the flask from the heat source and opening the reaction mixture to the atmosphere. To the solution was then added acetone (10 mL), after which the polymer was purified by 2-fold precipitation from cold diethyl ether and filtration. A white powder was obtained. **^1^H NMR** (400 MHz, CDCl_3_) δ 3.62–3.58 (m, 3 H), 1.81 (m, 2 H), 0.94 (m, 3 H). **IR***v* = 1730 cm^–1^ (—C(=O)—O), 1470–1430 cm^–1^ (−CH_3_), 1260 cm^–1^ (C–O), 1190–1150 cm^–1^ (C–O–C), 988 cm^–1^ (O–CH_3_).^66^

The polymerization of **TFEMA** was stopped after 24 h by removing the flask from the heat source and opening the reaction mixture to the atmosphere. To the solution was then added acetone (10 mL), after which the polymer was purified by 2-fold precipitation from cold diethyl ether and filtration. A white powder was obtained. **^1^H NMR** (400 MHz, CDCl_3_) δ 4.37 (s, 2 H), 2.00 (m, 2 H), 1.04 (m, 3 H). **IR***v* = 1743 cm^–1^ (—C(=O)—O), 1280 cm^–1^ (C–F), 655 cm^–1^ (C–F).^67,68^

The polymerization of **NMEP** was stopped after 6 h by removing the flask from the heat source and opening the reaction mixture to the atmosphere. To the solution was then added acetone (10 mL), after which the polymer was purified by 2-fold precipitation from cold diethyl ether and filtration. A white powder was obtained. **^1^H NMR** (400 MHz, CDCl_3_) δ 4.07 (s, 2 H), 3.54 (m, 4 H), 2.43 (s, 2 H), 2.12 (m, 2 H), 1.57 (s, 2 H), 0.96 (m, 3 H). **IR***v* = 1726 cm^–1^ (—C(=O)—O), 1664 cm^–1^ (—C(=O)—N), 1268 cm^–1^, 1424 cm^–1^ (C–H), 1267 cm^–1^ (C–N), 1147 cm^–1^ (C(=O)—O).^40,69^

### Modification of Gold, SiO_2_, and PE Substrates with Poly(dopamine)

1 × 1 cm substrates were rinsed using acetone, ethanol, and MilliQ and then dried under a gentle stream of argon. The substrates were placed in a Petri dish containing freshly prepared solution of dopamine.HCl (2 mg/mL) and NaIO_4_ (20 mM). The Petri dish was closed, sealed using Parafilm, and placed on an automated shaker at RT, 60 RPM. The surfaces were removed from the solution after the appropriate time, which was 10 min for gold, 20 min for silicon oxide, and 30 min for PE. They were then thoroughly rinsed with MilliQ and subsequently dried.

### Grafting of Polymers to Poly(dopamine)-Modified Substrates

The grafting-to reaction of the block copolymers was carried out according to the protocol described previously.^41^

### Gel Permeation Chromatography (GPC)

The polymer molecular weight and polydispersity index (PDI) were determined using gel permeation chromatography (Agilent 1200 Organic GPC + refractive index detector, equipped with PLgel 5 μm MIXED-D column). The column was calibrated with a poly(methyl methacrylate) polymer set. The selected eluent was THF for poly(GMA)_20_, poly(GMA)-*b*-poly(MMA), poly(GMA)-*b*-poly(MeOEGMA), and poly(GMA)-*b*-poly(TFEMA), and DMF + 0.1% LiBr for poly(GMA)-*b*-poly(NIPAM) and poly(GMA)-*b*-poly(NMEP), pumped at a constant flow of 0.5 mL/min.

### Ellipsometry

Ellipsometric angles Δ and Ψ of the synthesized polymer brushes were measured using an EP4 imaging ellipsometer (Accurion, Germany). The measurements were performed in air at room temperature in the wavelength range of λ = 491–761.3 nm at an angle of incidence of 50°. The acquired Δ and Ψ were fitted in the EP4 modeling software using a multilayer model to obtain dry polymer brush thickness and refraction index values. The poly(dopamine) layer was described using a Cauchy–Urbach model to account for the light absorption of poly(dopamine).
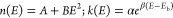


The following parameters were used: *A* = 1.54, *B* = 3000 nm^2^, α = 0.161 ± 0.58, β = 0.242 ± 0.121, and *E*_b_ = 3.0 eV. The poly(GMA)-*b*-poly(NIPAM) layer for polymer-modified substrates was described using a Cauchy model with parameters *A* = 1.50 and *B* = 3000 nm^2^. The poly(GMA)-*b*-poly(TFEMA) layer for polymer-modified substrates was described using a Cauchy model with parameters *A* = 1.41 and *B* = 3000 nm^2^. The poly(GMA)-*b*-poly(MMA) layer for polymer-modified substrates was described using a Cauchy model with parameters *A* = 1.49 and *B* = 3000 nm^2^. The poly(GMA)-*b*-poly(MeOEGMA) layer for polymer-modified substrates was described using a Cauchy model with parameters *A* = 1.45 and *B* = 3000 nm^2^. The poly(GMA)-*b*-poly(NMEP) layer for polymer-modified substrates was described using a Cauchy model with parameters *A* = 1.49 and *B* = 3000 nm^2^. Addition of an outermost layer to account for the roughness of the measured substrates using Bruggeman’s effective medium approximation (EMA) did not improve the fit of the model.^70^

### X-Ray Photoelectron Spectroscopy

XPS measurements were performed using a JPS-9200 photoelectron spectrometer (JEOL Ltd., Japan). All samples were analyzed using a focused monochromated Al Kα X-ray source (spot size of 300 μm) at a constant dwelling time for wide-scan 50 ms and a narrow-scan of 100 ms and pass energy: wide scan 50 eV, narrow-scan: 10 eV, under UHV conditions (base pressure: 3 × 10^–7^ Pa). The power of the X-ray source was 240 W (15 mA and 9 kV). Charge compensation was applied during the XPS scans with an accelerating voltage of 2.8 eV and a filament current of 4.8 A. All narrow-range spectra were corrected with a linear background before fitting. The spectra were fitted with symmetrical Gaussian/Lorentzian (GL(30)) line shapes using CasaXPS. The wide scan spectra and C 1s narrow scan spectra were referenced to the C 1s peak attributed to C–C and C–H atoms at 285.0 eV.

### ATR FT-IR Spectroscopy

FT-IR spectra were obtained on a Bruker Tensor 27 spectrometer with platinum-attenuated total reflection accessory. The samples were applied as powder or oil on top of the crystal. Sixty-four scans were performed with a resolution of 4 cm^–1^.

### Static Water Contact Angle Measurements

The wettability of the modified surfaces was determined by automated static water contact angle measurements using a DSA 100 goniometer (Krüss, Germany). The volume of a drop of demineralized water employed was 3 μL. Contact angles from sessile drops measured by the tangent method were estimated using a standard error propagation technique involving partial derivatives.

### Statistical Analysis

All statistical results were calculated using Origin 2019 software. Data were displayed as average values ± standard deviation.

## Results and Discussion

**Scheme 2:**
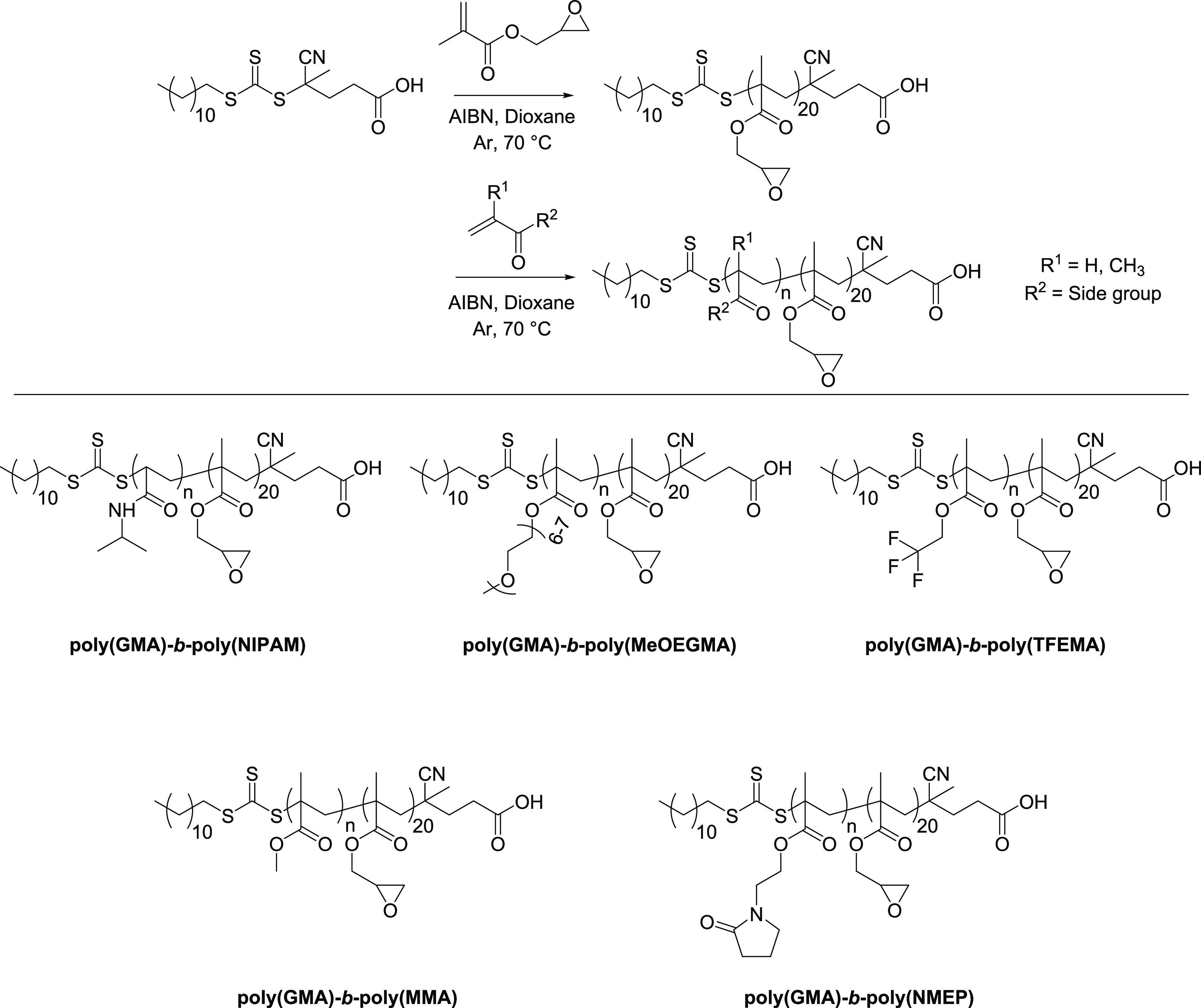
Molecular Structure of the Block Copolymers That Were Synthesized Using RAFT Polymerization and Employed in the Grafting-To Reactions in This Study

For this study, a library of five different diblock copolymers was synthesized via RAFT polymerization. Each block copolymer contains a segment of **GMA** units. The monomers that were used for the second segment differ for al block copolymers and were selected based on their distinct chemical properties as well as their wide-spread use in the field of polymer brush research (Scheme 2).

Methyl methacrylate (**MMA**) is one of the most commonly employed monomers in polymerization literature and widely used in day-to-day applications, such as dentures, acrylic glass, and electronics.^54^ (Oligo ethylene glycol) methyl ether methacrylate (**MeOEGMA**) was selected as it is the precursor to a hydrophilic polymer frequently used in antibiofouling surfaces.^55−57^ In contrast, trifluoroethyl methacrylate (**TFEMA**) is known to produce inert, hydrophobic polymer brushes that can be used to produce low-friction^58,59^ and antipolymer fouling surfaces.^60^ The *N*-isopropylacrylamide (NIPAM) monomer was included due to the well-known thermoresponsive character of poly(NIPAM).^61,62^ Similarly, (*N*-(2-methacryloyloxy)ethyl pyrrolidone (**NMEP**) was added due to its potential toward thermoresponsive, antifouling surfaces.^63−65^

Synthesis of the block copolymers was carried out in two steps (Scheme 2). First, **GMA** was polymerized to produce short polymer chains that contained approximately 20 repeating units (Table 1). Based on our recent work, both the nature and length of this **GMA** block is optimal for the grafting-to step by reaction with of the −NH_2_ moieties of the poly(dopamine)-modified substrates.^46,66,67^ Following the first polymerization step, the poly(GMA) was used as a macro-RAFT agent in a subsequent polymerization reaction with one of the aforementioned monomers. All polymerization reactions were carried out under identical conditions varying only in reaction time over a range from 4 to 48 h, to achieve polymer blocks with roughly 250 repeating units. The produced block copolymers were characterized using ^1^H NMR, GPC, and Fourier-transform infrared spectroscopy (FT-IR) (Table 1 and Supporting Information).

**Table 1:** Summary of the Block Copolymers Synthesized in This Study and Analyzed Using GPC

copolymer	*M*_n_ (kDa)	*M*_w_ (kDa)	*Đ*
poly(GMA)	3.6	4.6	1.27
poly(GMA)-*b*-poly(MMA)	31.5	39.9	1.27
poly(GMA)-*b*-poly(MeOEGMA)	59.5	89.7	1.51
poly(GMA)-*b*-poly(TFEMA)	43.4	55.7	1.28
poly(GMA)-*b*-poly(NIPAM)	28.5	37.2	1.30
poly(GMA)-*b*-poly(NMEP)	56.8	83.1	1.46

**Figure 1:**
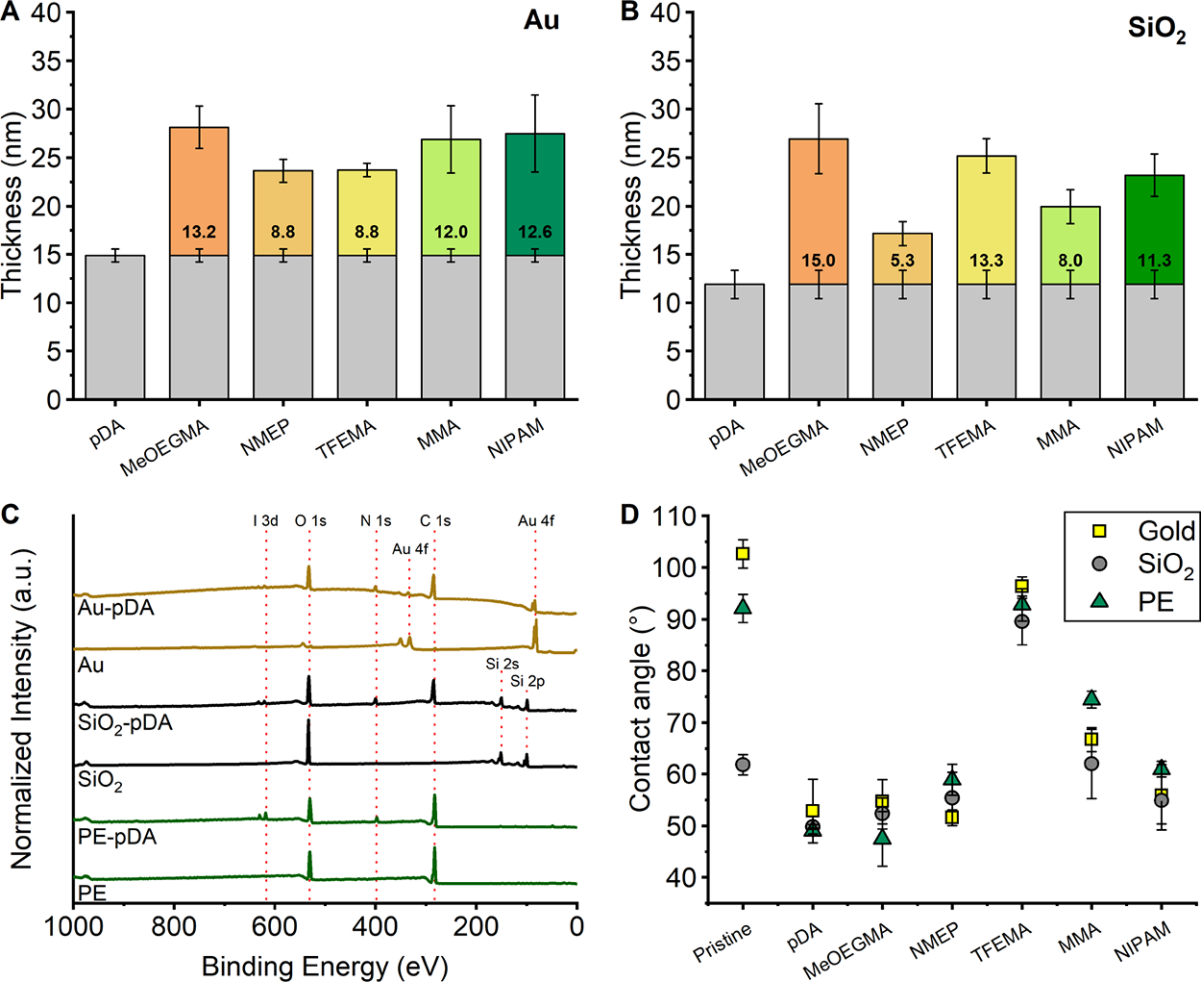
Layer thickness of poly(dopamine) film (gray bars) and block copolymers grafted to the poly(dopamine)-modified gold (**A**) and SiO_2_ (**B**) substrates determined by spectroscopic ellipsometry. (**C**) XPS wide scan spectra for pristine and poly(dopamine)-modified PE, SiO_2_, and gold samples. Note the appearance of the characteristic N 1s signal for the modified surfaces. (**D**) Static water contact angles for pristine substrates, poly(dopamine)-modified substrates, and poly(dopamine)-modified substrates after grafting of block copolymers.

Poly(dopamine) deposition was performed by dip-coating of the substrates in a solution of dopamine in NaOAc buffer (pH 7) in the presence of oxidizing agent NaIO_4_.^47^ The deposition reaction was performed until approximately 10 nm of poly(dopamine) could be measured on gold and SiO_2_ by spectroscopic ellipsometry (Figure 1A,B). For the PE substrates, the thickness of the poly(dopamine) layer could not be determined using spectroscopic ellipsometry as the applied film could not be differentiated from the polyester material underneath. Verification of the presence of poly(dopamine) on PE was therefore performed using XPS analysis (Figure 1C). After deposition, a distinct N 1s signal appeared in the XPS wide scan spectra of the PE substrates originating from the poly(dopamine) films. Likewise, the emergence of the nitrogen signal was observed for the gold and SiO_2_ substrates. Contact angle measurements were performed before and after poly(dopamine) deposition reactions (Figure 1D). Whereas the pristine samples exhibited large differences in contact angles, the poly(dopamine)-modified substrates all showed static water contact angles of approximately 50°, consistent with reported data in literature.^13^ These observations implied that the surface modifications on all three substrates yielded similar poly(dopamine) primer layers.

Following the poly(dopamine) depositions, the block copolymers were grafted to the modified substrates. The surfaces were submerged in solutions of the appropriate block copolymer in DMSO and heated to 80 °C overnight, together with triethylamine (5% v/v) to catalyze the reaction between the amine groups present in poly(dopamine) and the epoxides present in the block copolymers.^68^

After the grafting-to reactions, the layer thicknesses of the gold and SiO_2_ surfaces were determined using spectroscopic ellipsometry (Figure 1A,B). A substantial increase in layer thickness was observed for both surface types following attachment of the block copolymers. On the poly(dopamine)-modified gold substrates, the block copolymer layer thickness varied between roughly 9 and 13 nm. The average thickness increase was highly similar for the grafting reactions on poly(dopamine)-modified SiO_2_ substrates, albeit with a larger variance between samples. While not explicitly optimizing our conditions to obtain the highest grafting thickness, for the poly(GMA)-*b*-poly(MeOEGMA) block copolymers on SiO_2_ substrates, the highest layer thickness was achieved, reaching an average film thickness of already 15.0 ± 5.0 nm. Comparison of layer thicknesses on the different substrates did not reveal significant differences in grafting reactivity between the five block copolymers. It was therefore concluded that the reactivity toward grafting is similar for all block copolymers and the differences in observed layer thicknesses are the result of experimental variation.

Static water contact angles that are characteristic for the grafted polymers were observed for all substrates, irrespective of substrate nature (Figure 1D). Grafting of poly(GMA)-*b*-poly(MeOEGMA) and poly(GMA)-*b*-poly(NMEP) resulted in hydrophilic surfaces with average contact angles of 51 ± 4° and 55 ± 4°, respectively, which agrees with values reported in literature for poly(MeOEGMA) and poly(NMEP) brushes.^65,69,70^ Grafting of poly(GMA)-*b*-poly(TFEMA) and poly(GMA)-*b*-poly(MMA) resulted in more hydrophobic surfaces, with average water contact angles of 93 ± 3° and 68 ± 6°, respectively. These observations were in agreement with literature data for the polymer brush coatings of poly(TFEMA) and poly(MMA).^58,71,72^ Lastly, the grafting of thermoresponsive poly(GMA)-*b*-poly(NIPAM) resulted in an average water contact angle of 57 ± 3°, characteristic for surfaces modified with poly(NIPAM).^73^

**Figure 2:**
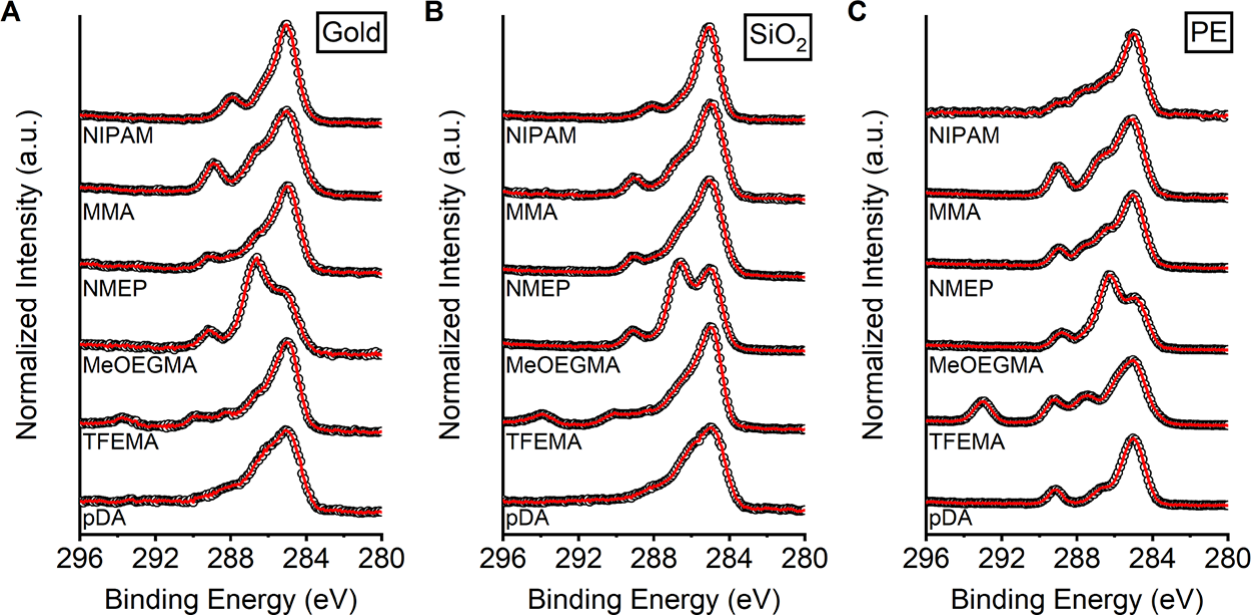
XPS C1s narrow scan spectra of gold (**A**), SiO_2_ (**B**), and PE (**C**) after modification with poly(dopamine) and after subsequent grafting of poly(GMA)-*b*-poly(TFEMA), poly(GMA)-*b*-poly(MeOEGMA), poly(GMA)-*b*-poly(NMEP), poly(GMA)-*b*-poly(MMA), and poly(GMA)-*b*-poly(NIPAM).

XPS measurements were performed to further characterize the produced surfaces and confirm the presence of the block copolymers on the substrates. Specifically, C 1s narrow scan spectra were collected to identify signals that are characteristic for the grafted block copolymers (Figure 2). In general, the spectra showed highly comparable results, irrespective of the substrate type. The spectra for poly(dopamine)-modified gold and SiO_2_ substrates were near-identical and were in agreement with binding energies found previously for poly(dopamine) films.^46,74,75^ The C1s spectrum for poly(dopamine)-modified PE displayed slightly different binding energies, which were attributed to electrons originating from the underlying polyester layer. Following grafting of poly(GMA)-*b*-poly(TFEMA) to the poly(dopamine) substrates, the emergence of a peak at 293.5 eV was observed, which is typical for the C–F bonds present in **TFEMA**.^60,76,77^ Additionally, the successful grafting of the block copolymer was confirmed by the emergence of the F 1s signal in the wide scan spectra (see the Supporting Information). The attachment of poly(GMA)-*b*-poly(MeOEGMA) was deduced from the appearance of strong signals at 286.5 eV that arise from the multiple C–O bonds present in the oligo(ethylene glycol) side group.^78^ The binding energies arising from attachment of poly(GMA)-*b*-poly(NMEP) were less distinct, although the presence of both the ester (—C(=O)—O) and cyclic amide (—C(=O)—N) could be observed by the increase in intensity at 289.0 and 288.0 eV, respectively.^65^ More apparent was the N 1s peak in the wide scan spectra, arising from the cyclic amide (see the Supporting Information). The substrates that were modified with poly(GMA)-*b*-poly(MMA) showed a distinct ester peak (—C(=O)—O) at 289.0 eV.^79,80^ Finally, the presence of poly(GMA)-*b*-poly(NIPAM) was confirmed via the presence of amide (—C(=O)—NH) signal in the C 1s spectra at 288.0 eV, as well as the strong N 1 s signal in the wide scan spectra (see the Supporting Information).

The analysis of the produced surfaces using spectroscopic ellipsometry, static water contact angle measurements, and XPS confirms that the two-step procedure employed in this study is both modular with respect to the substrate types employed here, as well as to the functional groups of the grafted polymers. Based on these results as well as previous studies that demonstrated successful adhesion of poly(dopamine) on a large range of substrates,^13,81^ it can be assumed that this grafting-to procedure can be easily applied to additional substrate types.

Since identical conditions were employed for the grafting reactions of all block copolymers, the method could potentially be used to synthesize binary brushes. Such coatings have become of increasing interest as they commonly exhibit switchable surface properties that can be applied in responsive coatings.^39,40,53,82−84^ The ability to easily synthesize such mixed polymer systems on any substrate would greatly enhance the applicability of responsive coatings.

**Scheme 3:**
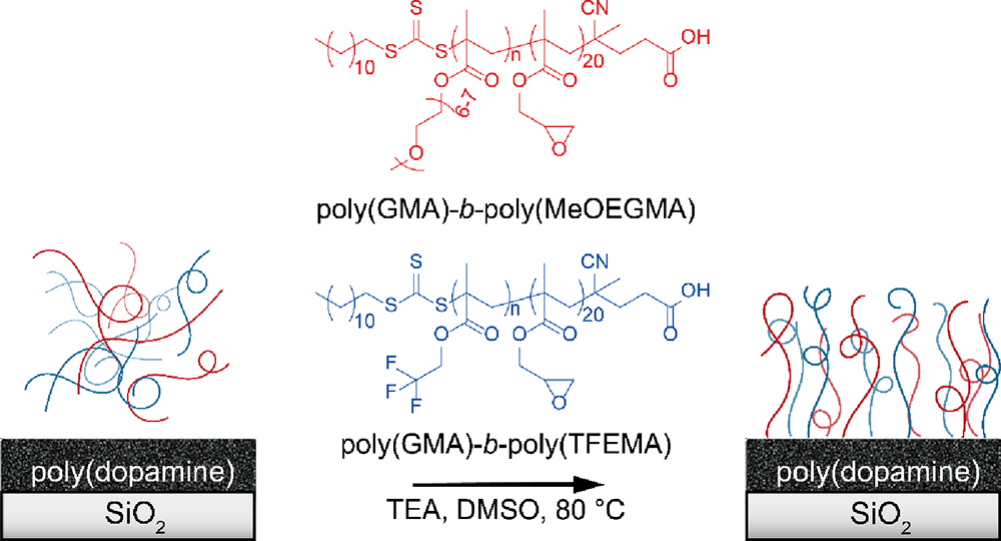
Illustration of the Synthesis of Binary Brush Systems Poly(GMA)-*b*-poly(TFEMA) and poly(GMA)-*b*-poly(MeOEGMA) copolymers, displayed as blue and red chains, respectively, were grafted to poly(dopamine)-modified SiO_2_ substrates.

To study the possible synthesis of binary brushes, grafting reactions using mixed polymer solutions of poly(GMA)-*b*-poly(TFEMA) and poly(GMA)-*b*-poly(MeOEGMA) were performed on poly(dopamine)-modified SiO_2_ (Scheme 3). The polymer mixtures were prepared with ratios poly(GMA)-*b*-poly(TFEMA) to poly(GMA)-*b*-poly(MeOEGMA) of 1:3, 1:1 and 3:1. The grafting step was carried out in the same manner as described for the single-block copolymer solutions.

**Figure 3:**
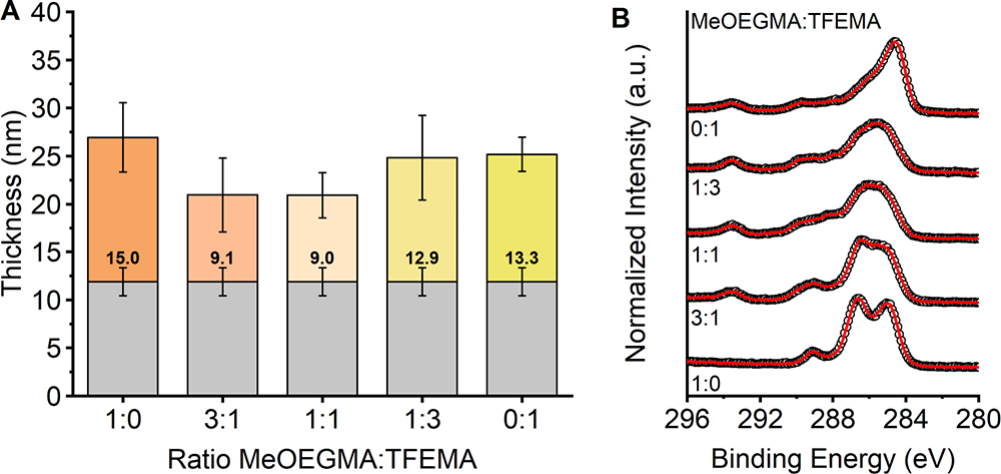
(**A**) Layer thickness of poly(dopamine) film (gray bars) and poly(GMA)-*b*-poly(MeOEGMA)/poly(GMA)-*b*-poly(TFEMA) binary brush systems on SiO_2_. (**B**) XPS carbon narrow scan spectra of poly(GMA)-*b*-poly(MeOEGMA)/poly(GMA)-*b*-poly(TFEMA) binary brushes grafted on poly(dopamine)-modified SiO_2_.

The thickness of the resulting coatings was determined using spectroscopic ellipsometry (Figure 3A). The layer of attached block copolymer was in the same thickness range as was previously observed for the single-polymer brushes. XPS C1s spectra were measured to confirm incorporation of both block copolymers during the grafting step (Figure 3B). Both block copolymers have distinctive binding energies originating from C–F at 293.5 eV in **TFEMA** and from C–O at 286.5 eV in **MeOEGMA**, which are apparent in the 0:1 and 1:0 spectra, respectively. In the spectrum of the 3:1 sample, signals at 293.5 and 286.5 eV can be seen clearly, indicating the presence of both the block copolymers on the surface. With increase in the relative amount of poly(GMA)-*b*-poly(TFEMA) with respect to poly(GMA)-*b*-poly(MeOEGMA), the intensity for the C–O signal at 286.5 eV decreases, whereas the intensity of the C–F at 293.5 eV in **TFEMA** increases. (Note: due to the underlying polydopamine layer, this trend is difficult to quantify, e.g., for proportionality between applied and immobilized ratios.) The carbon narrow scan spectra confirm that binary brushes can be synthesized using this simple procedure and that the ratio between the block copolymers on the surface can be easily tuned by variation of the ratio of block copolymers in the grafting solution. The ability to synthesize binary brushes with controlled composition enables the production of tunable responsive coating materials that exhibit, for example, switchable wetting behavior, morphology, and interactions with biological components.^41,42,82^

## Conclusions

We developed a surface-independent, easy-to-apply and modular two-step grafting-to procedure to synthesize polymer brushes. This yielded on gold, SiO_2_, and PE-coated glass grafted-to polymer brushes up to 15 nm, using a poly(dopamine) film as a primer layer. The grafting-to reactions were performed using block copolymers that carried a poly(GMA) segment, and allows freedom in the nature of the other segment of the copolymer, from hydrophobic to hydrophilic. Moreover, the method was used to synthesize binary brush systems by simply mixing in two-block copolymers in the grafting solution.

The exceptionally straightforward nature of the grafting reactions as well as its universal applicability in terms of the substrate type and polymer functionalities enables the introduction of wide range of surface properties to effectively any substrate type. The additional possibility to produce binary brush systems in a trivial manner gives this method further potential in the field of surface engineering.

## Supporting Information Available

GPC traces, FT-IR, and NMR spectra of the synthesized block copolymers as well as XPS wide scans for the produced polymer brush coatings (PDF)
