# Supplementary material for: Modular and Substrate-Independent Grafting-To Procedure for Functional Polymer Coatings
Source: Langmuir. 2023 May 22;39(22):7613–22. doi: 10.1021/acs.langmuir.3c00280 (PMC10249404; doi:10.1021/acs.langmuir.3c00280)
Supplement: Supplementary file 1 — la3c00280_si_001.pdf [file la3c00280_si_001.pdf]

Supporting Information to:

Modular and Substrate-Independent Grafting-to Procedure for Functional Polymer Coatings

Lucas W. Teunissen,[1] Maarten M. J. Smulders,[1] Han Zuilhof,[1,2,]*

1) Laboratory of Organic Chemistry, Wageningen University, Stippeneng 4, 6708 WE Wageningen, The Netherlands. 2) School of Pharmaceutical Sciences and Technology, Tianjin University, 92 Weijin Road, Tianjin, 300072, People's Republic of China.

Corresponding Author

E-mail: han.zuilhof@wur.nl

Table of contents

1. Supporting figures S2

1. Supporting Figures

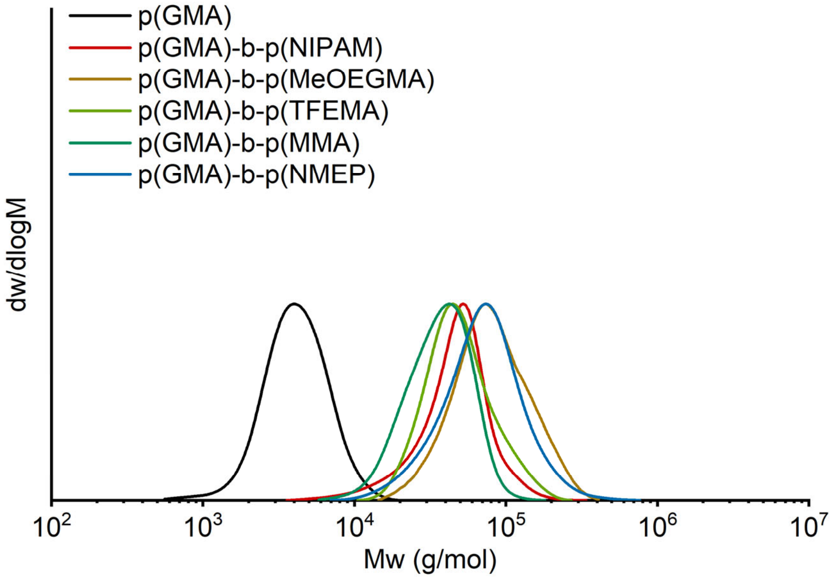

Figure S1. GPC traces for poly(GMA)$_{20}$ and block copolymers.

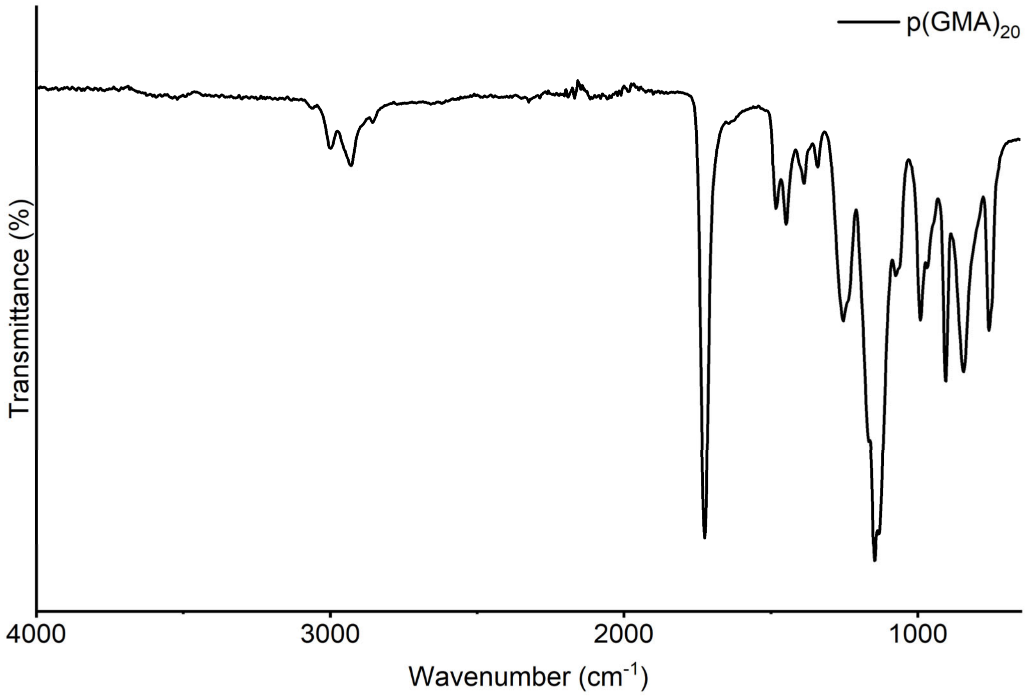

Figure S2. FT-IR spectrum of poly(GMA)$_{20}$.

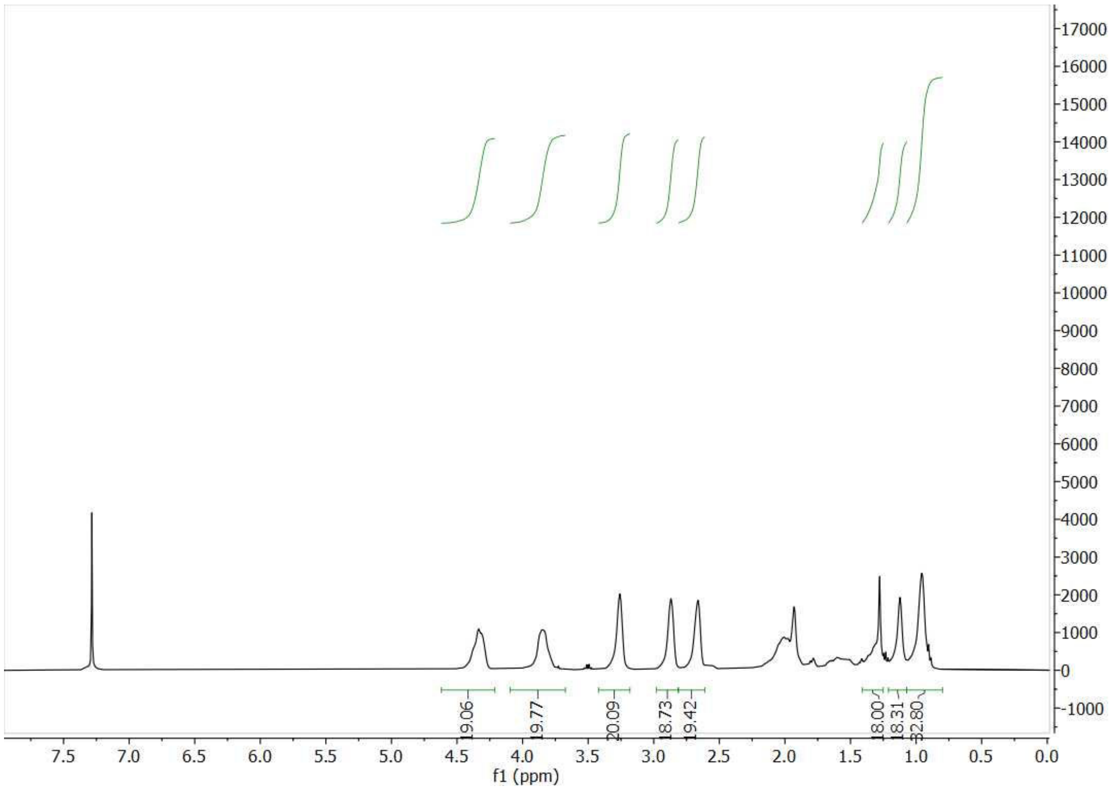

Figure S3. ^{1}H NMR spectrum of poly(GMA)$_{20}$ (in $CDCl_3$, 400 MHz, 298K).

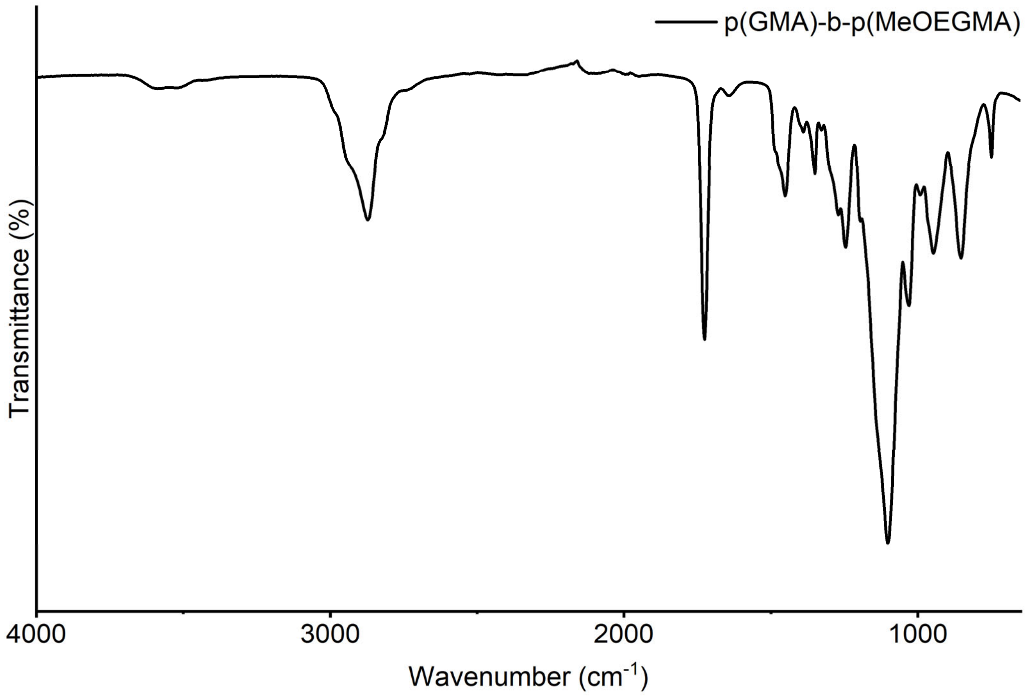

Figure S4. FT-IR spectrum of poly(GMA)-*b*-poly(MeOEGMA).

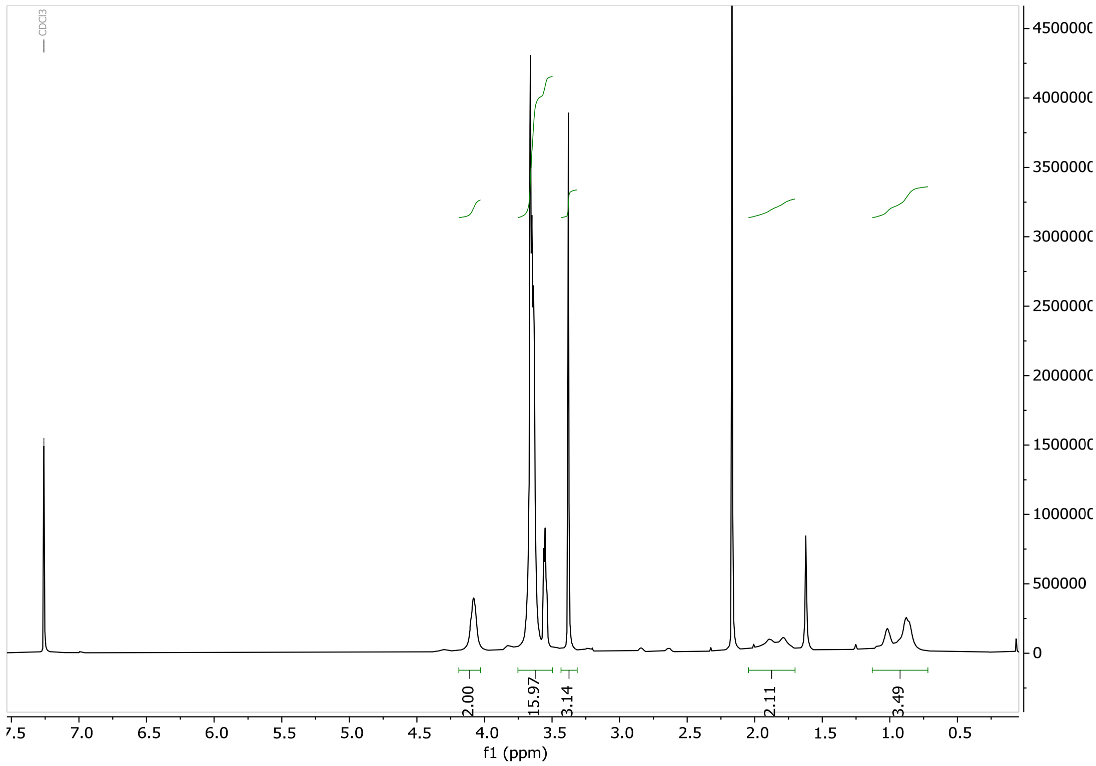

Figure S5. ^{1}H NMR spectrum of poly(GMA)-*b*-poly(MeOEGMA)

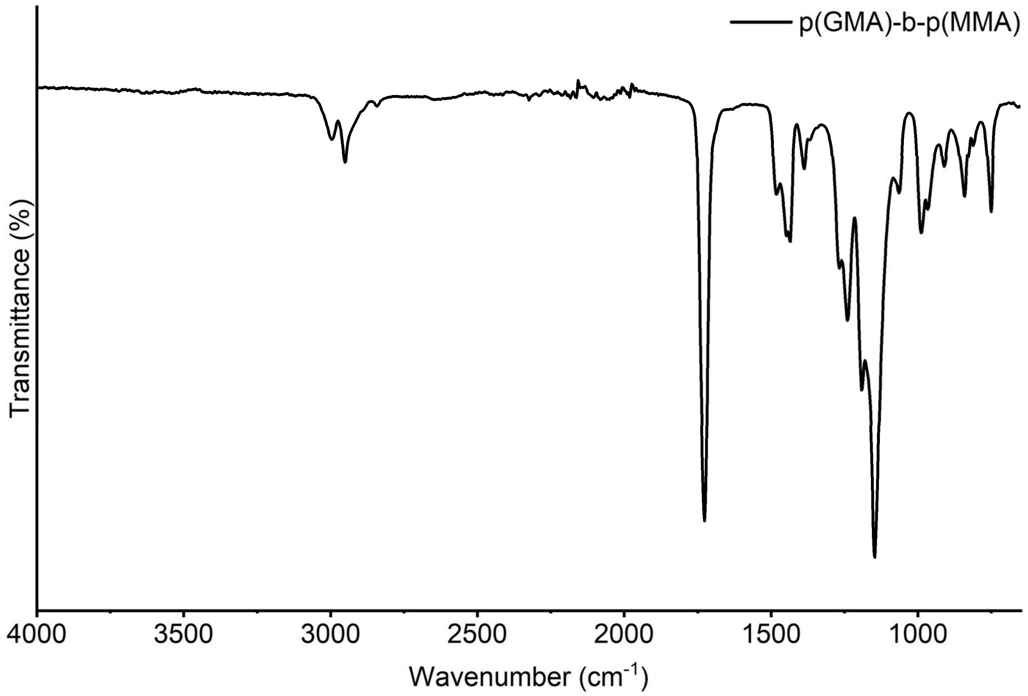

Figure S6. FT-IR spectrum of poly(GMA)-*b*-poly(MMA).

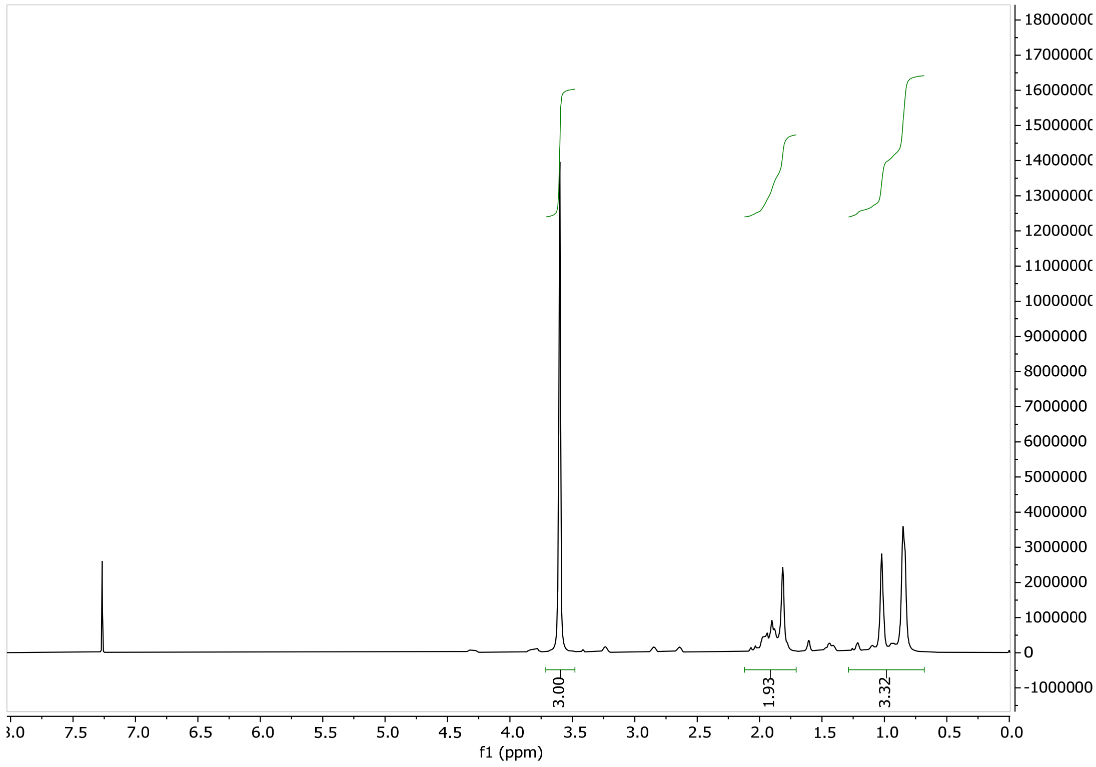

Figure S7. ^{1}H NMR spectrum of poly(GMA)-*b*-poly(MMA)

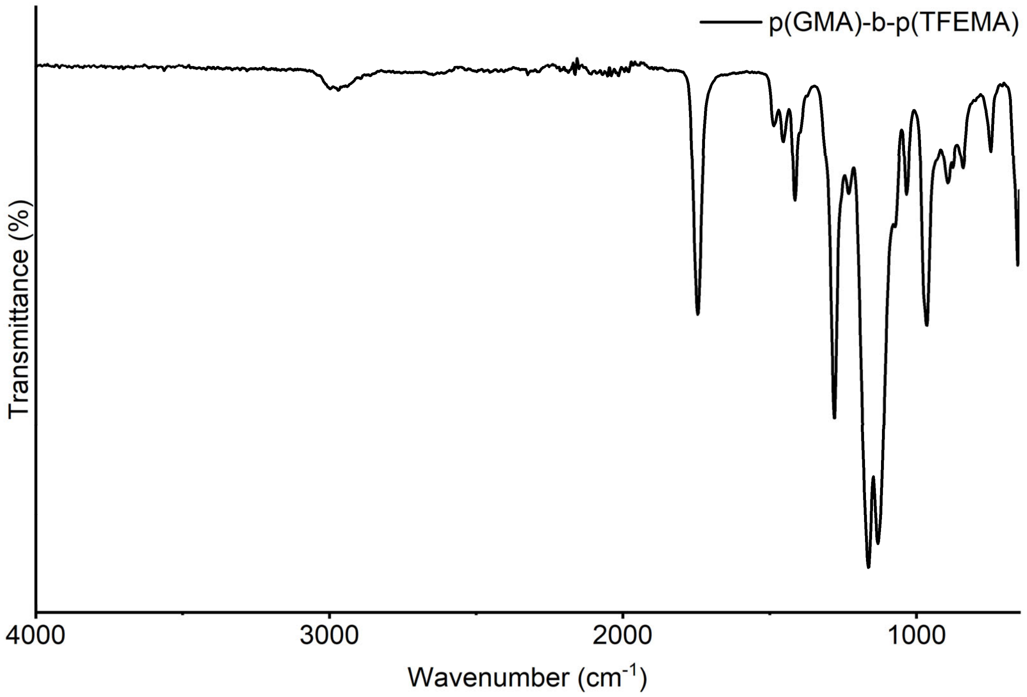

Figure S8. FT-IR spectrum of poly(GMA)-*b*-poly(TFEMA).

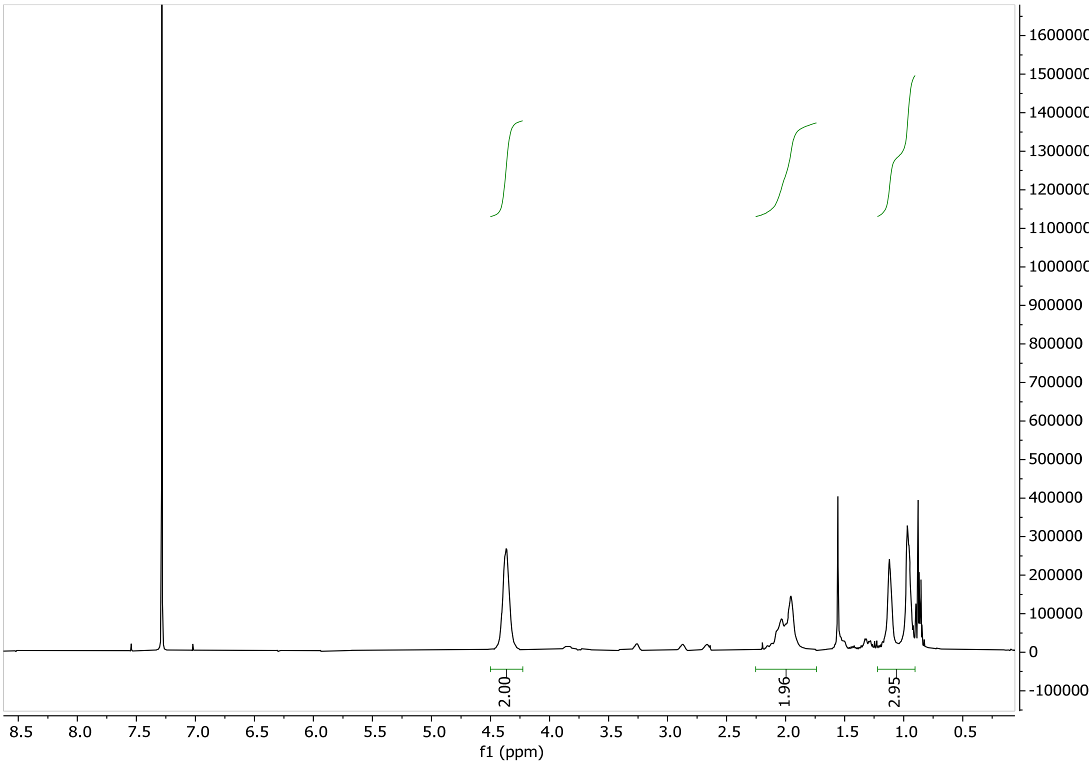

Figure S9. ^{1}H NMR spectrum of poly(GMA)-*b*-poly(TFEMA)

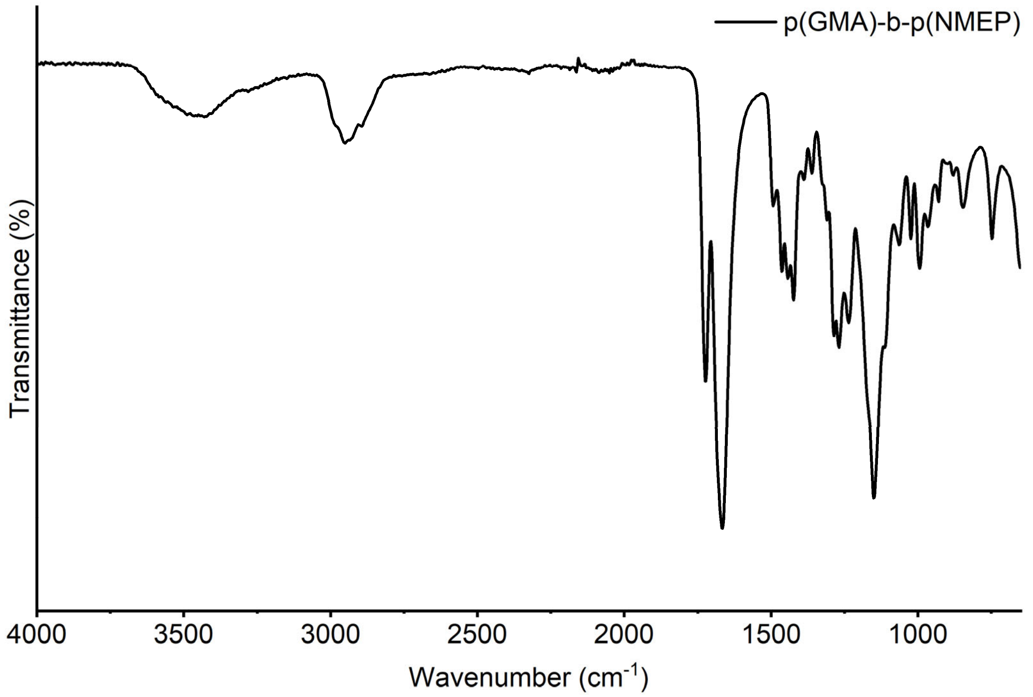

Figure S10. FT-IR spectrum of poly(GMA)-*b*-poly(NMEP).

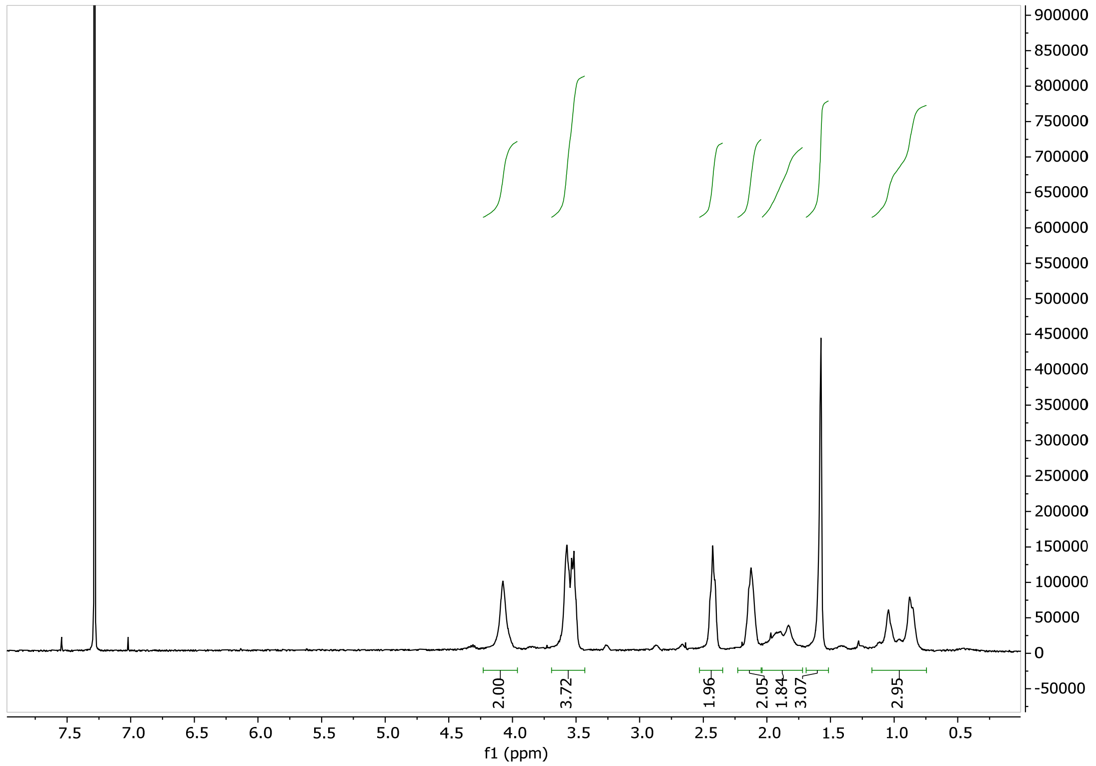

Figure S11. ^{1}H NMR spectrum of poly(GMA)-*b*-poly(NMEP)

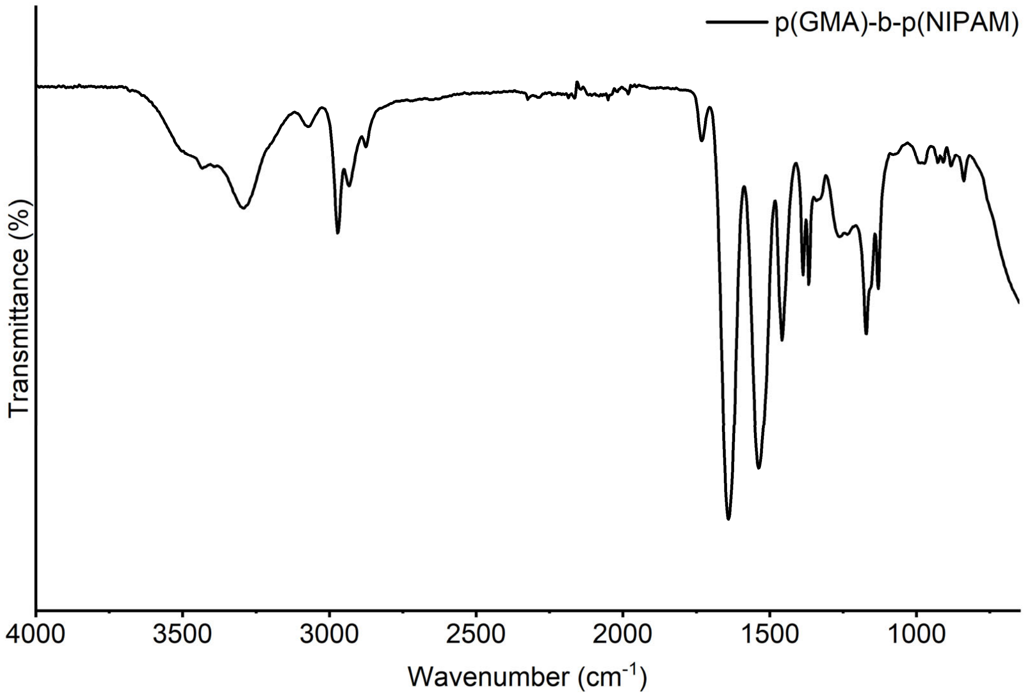

Figure S12. FT-IR spectrum of poly(GMA)-*b*-poly(NIPAM).

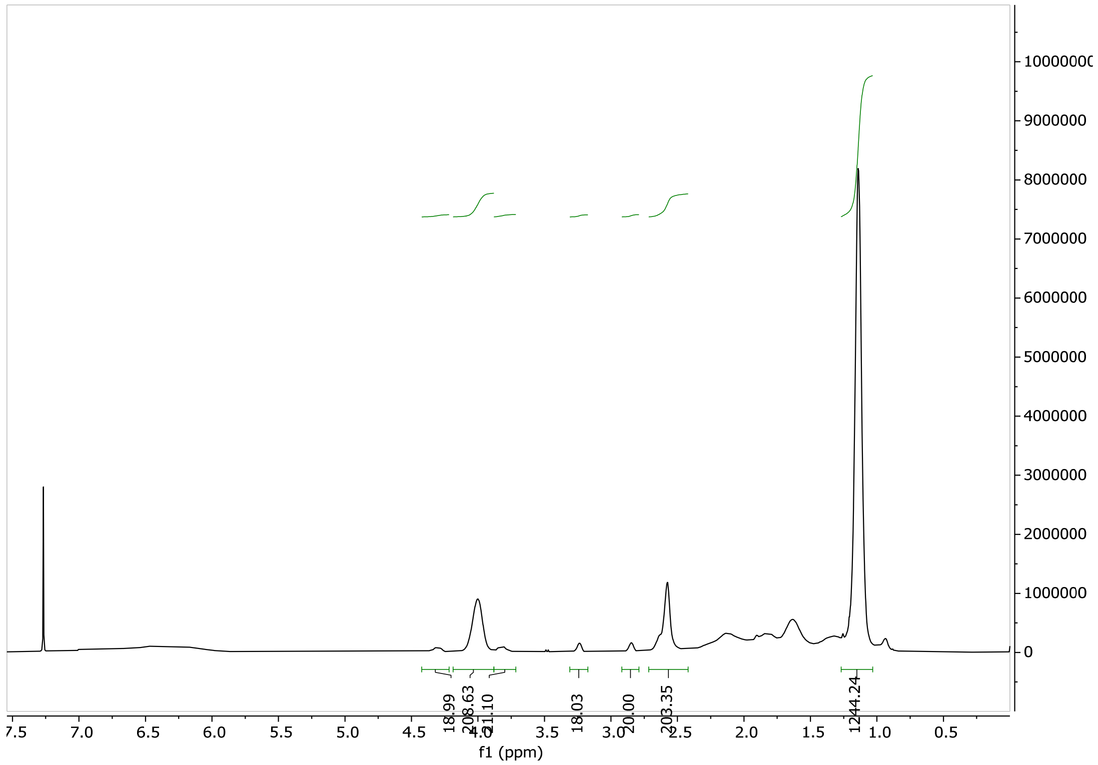

Figure S13. ^{1}H NMR spectrum of poly(GMA)-*b*-poly(NIPAM)

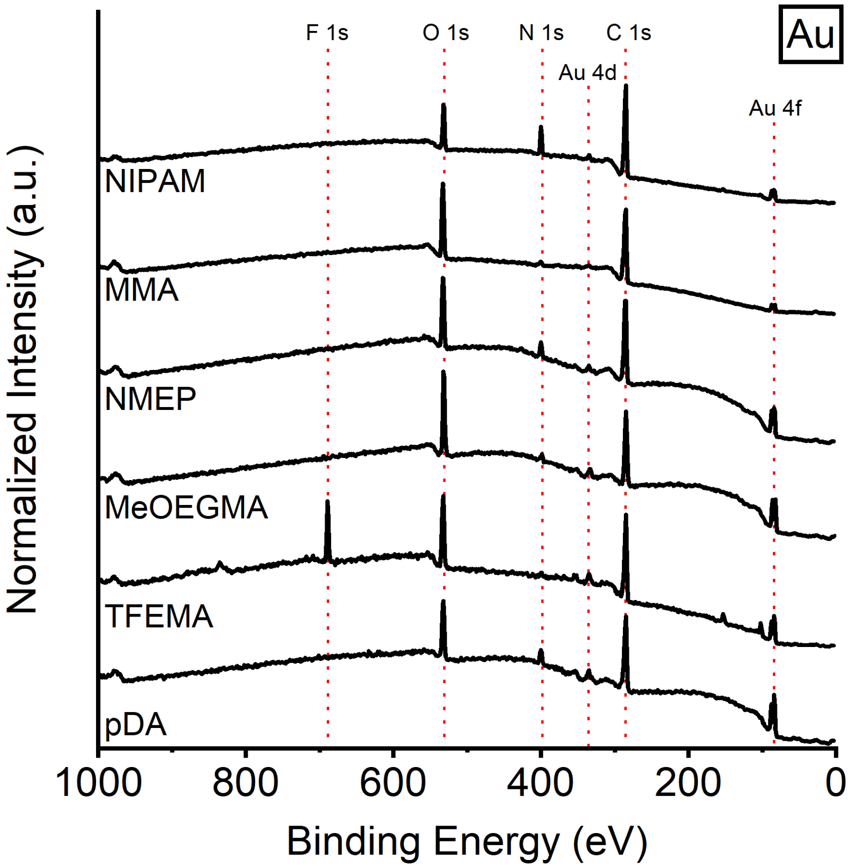

Figure S14. XPS wide scan spectra for gold substrates after modification with poly(dopamine) and after subsequent grafting of poly(GMA)-*b*-poly(TFEMA), poly(GMA)-*b*-poly(MeOEGMA), poly(GMA)-*b*-poly(NMEP), poly(GMA)-*b*-poly(MMA) and poly(GMA)-*b*-poly(NIPAM).

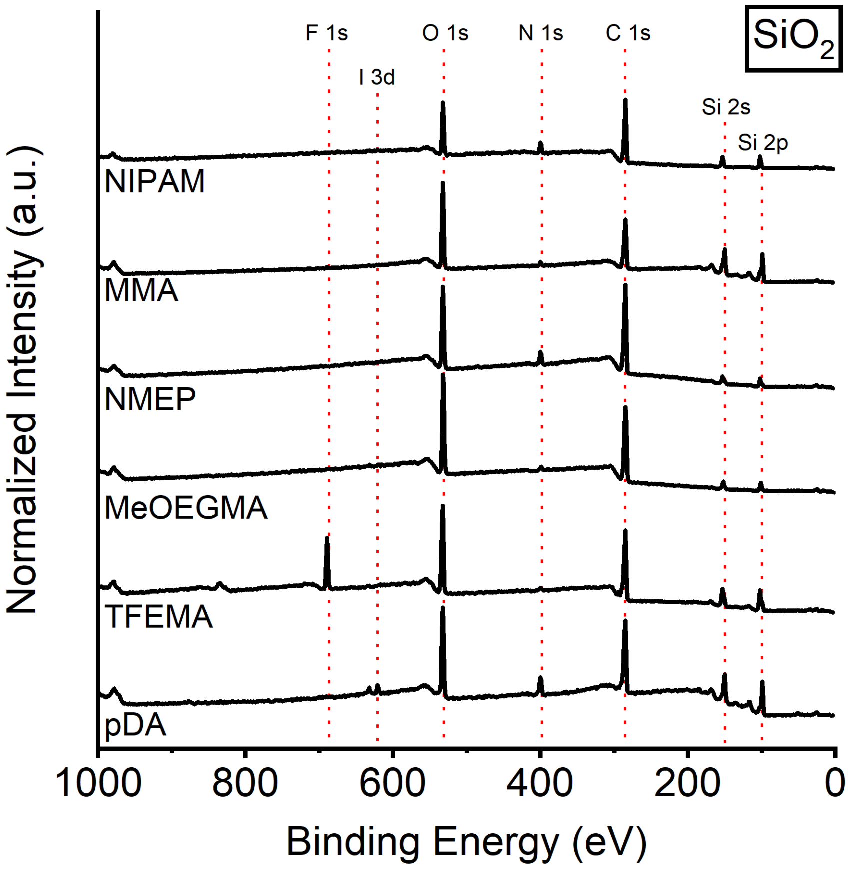

Figure S15. XPS wide scan spectra for SiO_2 substrates after modification with poly(dopamine) and after subsequent grafting of poly(GMA)-*b*-poly(TFEMA), poly(GMA)-*b*-poly(MeOEGMA), poly(GMA)-*b*-poly(NMEP), poly(GMA)-*b*-poly(MMA) and poly(GMA)-*b*-poly(NIPAM).

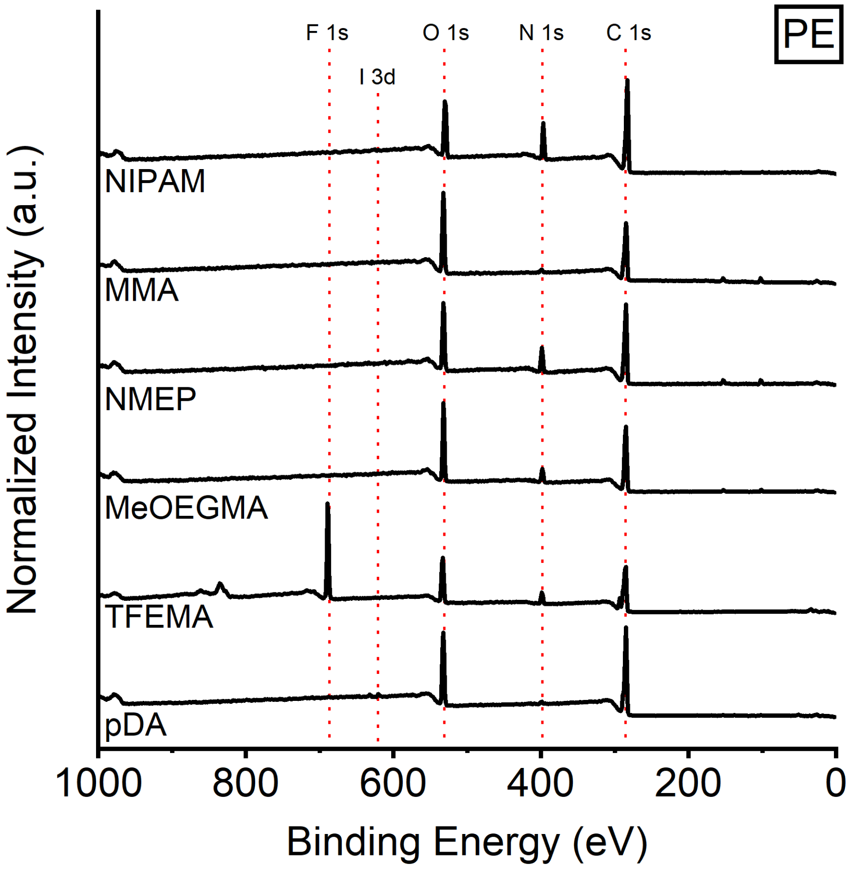

Figure S16. XPS wide scan spectra for PE substrates after modification with poly(dopamine) and after subsequent grafting of poly(GMA)-*b*-poly(TFEMA), poly(GMA)-*b*-poly(MeOEGMA), poly(GMA)-*b*-poly(NMEP), poly(GMA)-*b*-poly(MMA) and poly(GMA)-*b*-poly(NIPAM).

Note: The wide scan spectra presented for PE substrates modified with block copolymers poly(GMA)-*b*-poly(TFEMA) and poly(GMA)-*b*-poly(MeOEGMA) showed minor N 1s signals. A control experiment was performed in which poly(dopamine)-modified substrates are submerged in DMSO at 80 °C overnight without block copolymer or TEA (Figure S17). Still, the N 1s signal appears, which implies that the nitrogen-containing compound was already present within the PE polymer layer and had migrated towards the surface.

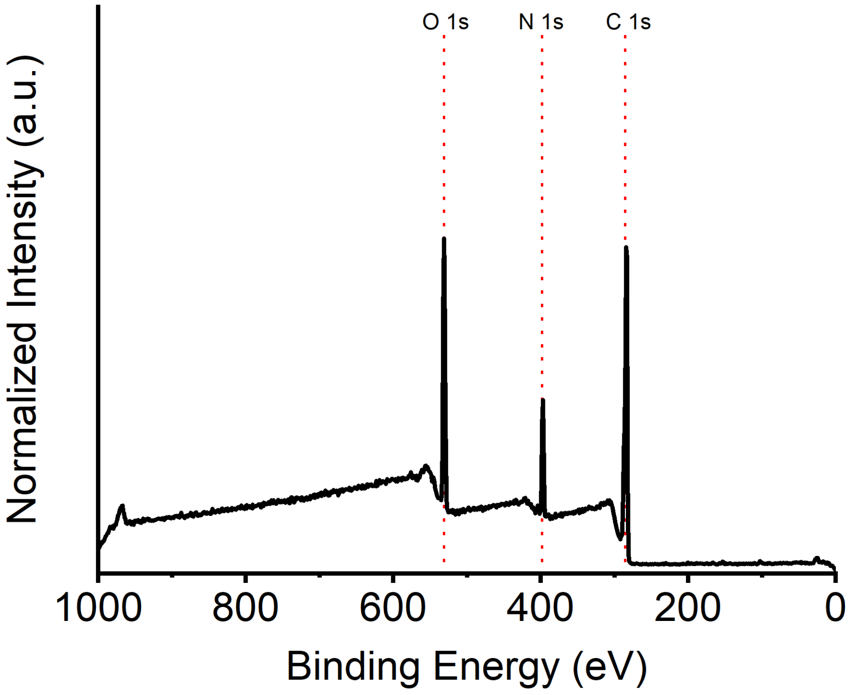

Figure S17. XPS wide scan spectra for control experiment involving immersion of PE substrate in DMSO overnight at 80 °C. The emergence of the N 1s signal implies that a nitrogen-containing compound was present within the PE polymer layer and had migrated towards the surface.
